# Heat-Killed and Live *Enterococcus faecalis* Attenuates Enlarged Prostate in an Animal Model of Benign Prostatic Hyperplasia

**DOI:** 10.4014/jmb.2102.02032

**Published:** 2021-06-29

**Authors:** Young-Jin Choi, Meiqi Fan, Yujiao Tang, Masahiro Iwasa, Kwon-Il Han, Hongchan Lee, Ji-Young Hwang, Bokyung Lee, Eun-Kyung Kim

**Affiliations:** 1 Department of Food Science and Nutrition, Dong-A University, Busan 49315, Republic of Korea; 2 Center for Silver-targeted Biomaterials, Brain Busan 21 Plus Program, Dong-A University, Busan 49315, Republic of Korea; 3 Division of Food Bioscience, College of Biomedical and Health Sciences, Konkuk University, Chungju 27478, Republic of Korea; 4 School of Bio-Science and Food Engineering, Changchun University of Science and Technology, Changchun 130-600, P.R. China; 5 R&D Center, Korea BeRM Co., Ltd., Wonju 26362, Republic of Korea; 6 Wiebien Hospital, Seoul 06035, Republic of Korea; 7 Department of Food Science and Technology, Dong-Eui University, Busan 47340, Republic of Korea

**Keywords:** Benign prostatic hyperplasia, *Enterococcus faecalis*, heat-killed microorganism

## Abstract

In the present study, we investigated the inhibitory effect of heat-killed *Enterococcus faecalis* (*E. faecalis*) and live *E. faecalis* on benign prostatic hyperplasia (BPH). The BPH rat model was established by administering male rats with testosterone propionate (TP, 5 mg/kg, in corn oil) via subcutaneous injections daily for four weeks after castration. The rats were divided into five groups: Con, corn oil-injected (s.c.) + DW administration; BPH, TP (5 mg/kg, s.c.) + DW administration; BPH+K_EF, TP (5 mg/kg, s.c.) + heat-killed *E. faecalis* (7.5 × 10^12^ CFU/g, 2.21 mg/kg) administration; BPH+L_EF, TP (5 mg/kg, s.c.) + live *E. faecalis* (1 × 10^11^ CFU/g, 166 mg/kg) administration; BPH+Fi, TP (5 mg/kg, s.c.) + finasteride (1 mg/kg) administration. In both of BPH+K_EF and BPH+L_EF groups, the prostate weight decreased and histological changes due to TP treatment recovered to the level of the Con group. Both of these groups also showed regulation of androgen-signaling factors, growth factors, and apoptosis-related factors in prostate tissue. *E. faecalis* exhibited an inhibitory effect on benign prostatic hyperplasia, and even heat-killed *E. faecalis* showed similar efficacy on the live cells in the BPH rat model. As the first investigation into the effect of heat-killed and live *E. faecalis* on BPH, our study suggests that heat-killed *E. faecalis* might be a food additive candidate for use in various foods, regardless of heat processing.

## Introduction

Benign prostatic hyperplasia (BPH) is considered an inevitable aging-associated disease prevalent among men. Similar to other chronic diseases, BPH is caused by many complex risk factors [1]. The pathology of BPH includes abnormal symptoms of androgen-related prostate cells and impaired cell death [2]. Despite the medical significance of BPH in aged men, the pathogenesis of this disorder is unclear. However, theories regarding imbalance of androgen and estrogen [3], oxidative stress [4], metabolic syndrome [5], inflammation [6], and autonomic nerves [7] have been implicated.

BPH is considered to cause hyperplasia of prostate cells because of the production of excessive dihydrotestosterone (DHT) [8]. In prostate cells, testosterone, a typical androgen secreted by testicular Leydig cells, is converted to DHT by 5α-reductase type 2 (5AR2) [9]. DHT binds to the androgen receptor (AR) or estrogen receptor (ER) in the prostate cells of BPH. A gradual decrease in the secretion of testosterone with age leads to an imbalance in the endocrine system. The consequent increase in DHT-receptor interaction results in an increase in the androgen receptor in prostate cells [10]. Moreover, chronic inflammation of the prostate is closely related to prostatic hyperplasia [11]. Tissue repair and inflammatory responses could result in thickening of the prostate tissue, and the resulting chronic inflammation leads to abnormal growth of the prostate gland. The increase in the levels of inflammatory cytokines induces the secretion of growth factors [12], which along with the recovery of damaged tissues, leads to abnormal prostate tissue proliferation. Additionally, BPH is characterized by activation of extracellular signal-regulated kinase (ERK). While ERK molecules are generally accumulated in the nucleus of normal prostate cells, they are secluded from the cytoplasm and are bound to the N-terminus of MEK in the prostate cells of BPH. Thus, increase in the active level of ERK results in continuous cell growth and inhibition of apoptosis in the prostate cells of BPH [13].

Recent studies on new therapeutic approaches for BPH have focused on in vitro and in vivo immune regulation functions of probiotics, heat-killed probiotics, and metabolites. A few studies have shown that killed probiotics are more stable and easier to use [14]. Recently, killed probiotics have been recognized as a better source of raw materials for natural products than live probiotics. Moreover, the potential applications of killed probiotics in general foods, functional foods, pharmaceuticals, and feeds have been steadily expanding [15]. *Enterococcus faecalis* (*E. faecalis*) is an anaerobic gram-positive bacterium that exhibits immunomodulatory activity [16]. It is a biogenic lactic acid bacterium that is used as a biological response modifier (BRM). *E. faecalis* can be heat-treated to produce a BRM containing high levels of β-glucan, which has shown excellent ameliorative effects on chronic and inflammatory diseases. Previous studies have reported the effects of heat-killed *E. faecalis* in allergic dermatitis [17, 18], muscle atrophy [19], inflammatory bowel disease [20] and tumor [21]. However, no study has been conducted on the BPH model so far. In addition, no studies of BPH using probiotics have been reported. Thus, in this study, the effects of heat-killed *E. faecalis* (K_EF) and live *E. faecalis* (L_EF) on BPH were investigated.

## Materials and Methods

### Chemicals

Testosterone propionate (TP) was provided by Tokyo Chemical Industry Co. (Japan). Finasteride (Fi) DHT, radioimmunoprecipitation assay (RIPA) buffer, and protease inhibitor cocktail were purchased from Sigma-Aldrich Inc. (USA). Aspartate aminotransferase (AST) and alanine aminotransferase (ALT) enzyme-linked immunosorbent assay (ELISA) kit was purchased from MyBioSource, Inc. (USA). DHT ELISA was purchased from SunLong Biotech Co. (China).

### *E. faecalis* Preparation

K_EF and live L_EF were supplied as dried powder by Korea BRM Co., Ltd. (Korea). One gram of dried L_EF contained 1 × 10^11^ bacteria. An equivalent amount of K_EF contained 7.5 × 10^12^ bacteria. *E. faecalis* was originally isolated from healthy human feces, and K_EF was supplied as a patented heat-killed preparation.

### Animals

Eight-week-old Sprague-Dawley male rats (*n* = 40) were purchased from Nara Biotech Ltd. (Korea), and the temperature of the breeding room was 23 ± 2°C. The relative humidity was maintained at 50 ± 10%, and food and water were freely ingested. All animal care and experimental procedures were approved by the Konkuk University Institutional Animal Care and Use Committee (KU20017).

### Animal Study Design

Animal experiments were conducted as reported previously [22]. Castration was performed to suppress the intrinsic testosterone produced in the testes [23]. Rats in BPH-induced groups were anesthetized with an intraperitoneal injection of phenobarbital (50 mg/kg). Both testicles were castrated aseptically. After castration, the rats stabilized for 3 days. At this time, the sutured areas of the mice were sterilized daily with povidone-iodine. BPH-induced groups were subcutaneously injected (s.c.) with TP (5 mg/kg/d) dissolved in commercially available corn oil. The BPH model was induced by TP administration for 28 consecutive days. *E. faecalis* at dietary concentration of 1×10^13^ CFU/60 kg/d was orally administered to rats. A suspension of heat-killed *E. faecalis* (1.66× 10^12^ CFU/kg/d) or live *E. faecalis* (1.66 × 10^12^ CFU/kg/d) in distilled water was orally administered to rats in the respective group. Fi (1 mg/kg/d) dissolved in DW was administered orally and used as a positive control. After 4 weeks of treatment, rats were anesthetized with pentobarbital (50 mg/kg, i.p.). Blood samples were collected via cardiac bleeding. All lobes of the prostate tissue were then excised and weighed. The ventral lobe of the prostate sections was fixed with 10% formaldehyde for histological analysis, and a portion of the ventral lobe was cut using a scalpel, then quickly frozen in liquid nitrogen and stored in a deep freezer for western blot analysis.

### DHT ELISA

Serum from the rats was obtained by centrifuging the blood at 1,000 ×*g* for 20 min at 4°C. Serum was stored at -80°C until analysis. The concentration of DHT in the serum and prostate tissue was determined using a DHT ELISA kit according to the manufacturer’s instructions. Optical density was measured at 450nm wavelength using a spectrophotometer.

### Serum Concentrations of AST and ALT

To verify liver toxicity, AST and ALT content in the serum of rats were measured using an ELISA kit. After conducting the experiment according to the method suggested by the ELISA kit manufacturer, optical density was measured and analyzed at 450 nm wavelength using a spectrophotometer.

### Hematoxylin and Eosin (H & E) Staining

The ventral lobe of the prostate fixed with 10% formaldehyde was dehydrated and embedded in paraffin. The prostate tissue fixed in paraffin was cut to a thickness of 4 μm, and then the paraffin was removed with xylene and reduced by successive treatment with alcohol. For H&E staining, the tissue was immersed in hematoxylin solution for 5 min, distilled water for 5 min, and eosin solution for 30 s, followed by drying and fixing. Prostate epithelial thickness and density were measured using Image J 1.47v software (National Institute of Health, USA).

### Western Blotting

Prostate tissues were homogenized by using the taco Prep Bead Beater (GeneReach, Taiwan). Homogenized tissues were lysed in cold RIPA buffer (mixed protease inhibitor cocktail) and centrifuged at 13,000 ×*g* for 20 min at 4°C. Concentration of extracted proteins was measured by using BCA assay. Cell lysates (50 μg/ml) were separated by 10% sodium dodecyl sulfate-polyacrylamide gel electrophoresis (SDS-PAGE) and transferred onto nitrocellulose membranes (0.45 μm pore size). The membranes were blocked with 5% (w/v) skim milk in TBST for 1 h at room temperature. The membranes were then immediately incubated with primary antibodies (1:1000 dilution) against β-actin (Santa Cruz Biotechnology, USA), 5AR2 (Abcam Inc., USA), ER (Cell Signaling Technology, Inc., USA), AR (Santa Cruz Biotechnology), PSA (Santa Cruz Biotechnology), VEGF (Santa Cruz Biotechnology), EGF (Santa Cruz Biotechnology), IGF-1 (Abcam Inc.), extracellular signal-regulated kinase (ERK; Cell Signaling Technology), p-ERK (Cell Signaling Technology), proliferating cell nuclear antigen (PCNA)(Santa Cruz Biotechnology), Cyclin D1 (Cell Signaling Technology), Bcl-2 (Cell Signaling Technology), or Bax (Cell Signaling Technology) overnight at 4°C. Membranes were incubated with HRP-conjugated anti-mouse IgG (Cell Signaling Technology), anti-rabbit IgG (Cell Signaling Technology) or anti-goat IgG (Santa Cruz Biotechnology) for 2 h at room temperature. Subsequently, membranes were photographed using an Azure C300 Imaging System (Azure Biosystems, USA). The chemiluminescence intensities of the protein band signals were quantified using NIH ImageJ 1.47v software.

### Statistical Analysis

All data are presented as mean ± standard error of the mean (SEM) derived from three independent experiments. Statistical significance was analyzed by one-way analysis of variance (ANOVA) or one-tailed Student’s t-test using IBM SPSS Statistics 22 software (International Business Machines Corp., USA). *p* < 0.05 and *p* < 0.01 were considered to denote statistical significance.

## Results

### Effect of *E. faecalis* on Prostate Weight and Prostate Index

**Fig. 1:**
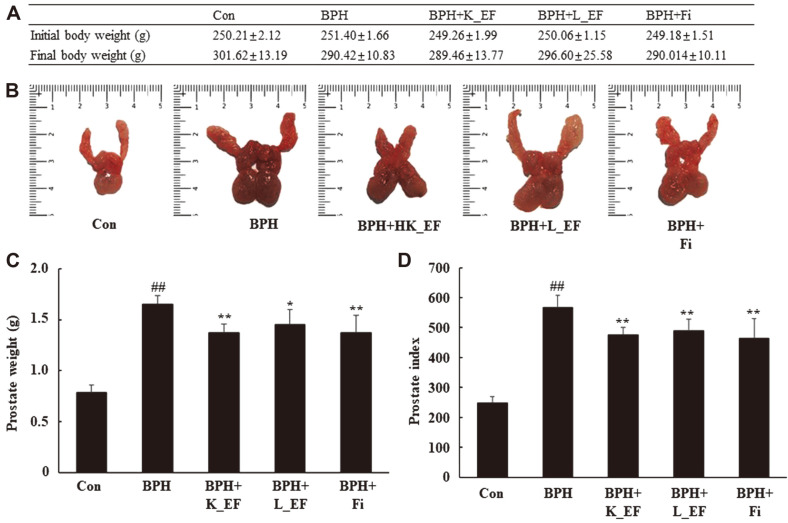
Change in prostate weight and prostate index following administration of K_EF and L_EF in BPH. (**A**) Body weight of the rats. (**B**) Prostate tissue of rats. (**C**) Prostate weight of the rats. (**D**) Prostate index. Con, corn oil-injected (s.c.) + DW administration; BPH, TP (5 mg/kg, s.c.) + DW administration; BPH+K_EF, BPH+ heat-killed *E. faecalis* (7.5 × 10^12^ CFU/g, 2.21 mg/kg) administration; BPH+L_EF, BPH + live *E. faecalis* (1 × 10^11^ CFU/g, 166 mg/kg) administration; BPH+Fi, BPH + finasteride (1 mg/kg) administration. Data are expressed as mean ± S.E.M. (*n* = 8). ^##^*p* < 0.01 compared to Con. *p < 0.05, **p < 0.01 compared to BPH.

The potential therapeutic effects of *E. faecalis* were investigated in the TP-induced BPH rat model. The prostate of a rat is composed of the dorsolateral prostate lobe (DLP), ventral prostate lobe (VP) and anterior prostate lobe (AP). Following 28-day treatment, the prostate glands of rats in the five groups were analyzed. The three prostate tissues (DLP, VP, and AP) were isolated, photographed, and weighed. The body weights of the BPH group were lower than those in the Con group but was not a significant difference (Fig. 1A). The prostate index of BPH group showed a significant difference by 2.29 times compared to the Con group. The prostate index in the BPH + K_EF and BPH + L_EF groups was significantly reduced by 17.1% and 13.7%, respectively, compared to the BPH group. Similarly, the positive control, BPH+Fi group, showed a significant decrease of 18.2% compared to the BPH group.

### Effect of *E. faecalis* on Histology of Ventral Prostate

**Fig. 2:**
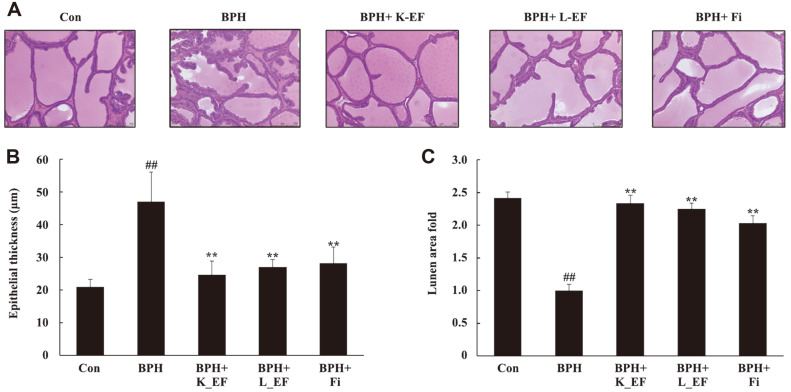
Effect of K_EF and L_EF on the histology of ventral prostate in BPH (**A**) H&E-stained prostate tissue from rats (magnification, X100). (**B**) The epithelial thickness of the prostate tissues from rats. (**C**) Lumen area fold in the prostate tissues from rats. Con, corn oil-injected (s.c.) + DW administration; BPH, TP (5 mg/kg, s.c.) + DW administration; BPH+K_EF, BPH+ heat-killed *E. faecalis* (7.5 × 10^12^ CFU/g, 2.21 mg/kg) administration; BPH+L_EF, BPH + live *E. faecalis* (1 × 10^11^ CFU/g, 166 mg/kg) administration; BPH+Fi, BPH + finasteride (1 mg/kg) administration. Data are expressed as mean ± S.E.M. (*n* = 8). Significant differences at ^##^*p* < 0.01 compared to Con. **p < 0.01 compared to BPH.

Fig. 2 shows epithelial thickness and lumen area in the ventral prostate lobe of rats using H & E staining. In BPH, prostate cells became enlarged and the number of cells increased, leading to an increase in epithelial thickness. Histological analysis of the prostate tissue showed a normal cell morphology for the Con group (Fig. 2A). On the other hand, in the BPH group, the epithelial thickness of the prostate was significantly increased compared to the Con group (Fig. 2B). Heat-killed *E. faecalis*, live *E. faecalis*, or Fi treatment reduced epithelial thickness in BPH group. Likewise, lumen area of In BPH+K_EF, BPH+L_EF, and BPH+Fi groups was significantly increased compared to the BPH group (Fig. 2C).

### Effect of *E. faecalis* on DHT Levels and Hepatotoxicity

**Fig. 3:**
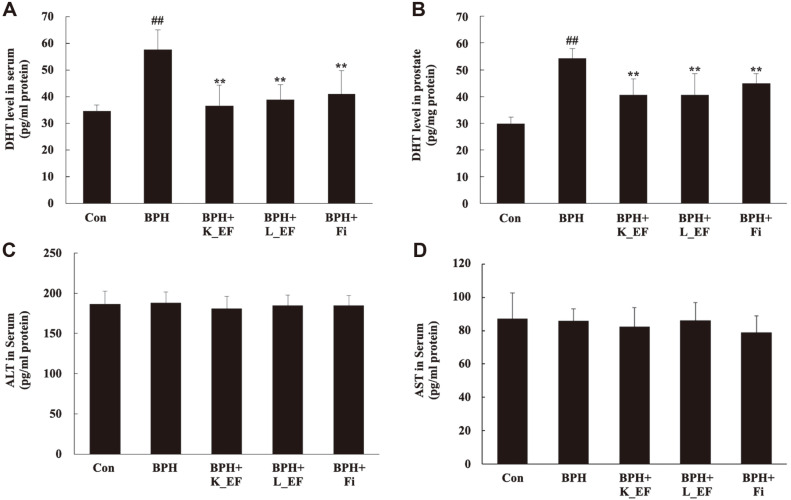
Biochemical analysis on serum and prostate tissue from BPH rats. (**A**) DHT level in serum. (**B**) DHT level in the prostate tissues. (**C**) ALT level in the serum. (**D**) AST level in the serum. Con, corn oil-injected (s.c.) + DW administration; BPH, TP (5 mg/kg, s.c.) + DW administration; BPH+K_EF, BPH+ heat-killed *E. faecalis* (7.5 × 10^12^ CFU/g, 2.21 mg/kg) administration; BPH+L_EF, BPH + live *E. faecalis* (1 × 10^11^ CFU/g, 166 mg/kg) administration; BPH+Fi, BPH + finasteride (1 mg/kg) administration. Data are expressed as mean ± S.E.M. (*n* = 8). ^##^*p* < 0.01 compared to Con. **p < 0.01 compared to BPH.

Fig. 3 shows concentration of DHT in serum or prostate tissue of rats. DHT levels in serum or prostate tissue were significantly higher in the BPH group compared to the Con group. The levels of DHT in the BPH + K_EF, BPH + L_EF, and BPH + Fi groups were significantly decreased compared to the BPH group (*p* < 0.05). Moreover, the DHT level of BPH+K-EF was the lowest among all groups. The liver has a very high concentration of enzymes and a blood circulation structure that allows easy leakage into the blood. Therefore, measuring the activity of liver enzymes released into the blood from a damaged liver is one of the most useful methods in liver toxicity studies. In particular, enzymes such as AST and ALT show high activity as hepatocyte necrosis and liver tissue destruction proceed [24]. Heat-killed *E. faecalis* or live *E. faecalis* treatment did not produce any toxicity.

### Effect of *E. faecalis* on the Expression of AR-Signaling-Related Genes

**Fig. 4:**
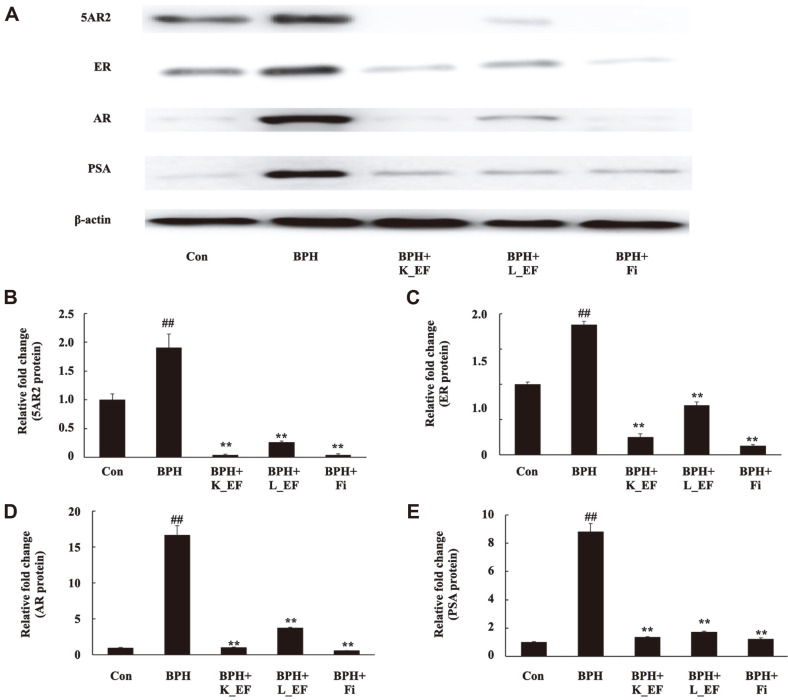
Effect of K_EF and L_EF on the expression of AR signaling-related factors in prostate tissue from BPH rats. (**A**) Protein expression of 5AR2, ER, AR, and PSA. Densitometer analysis of (**B**) 5AR2 expression, (**C**) ER expression, (**D**) AR expression, and (**E**) PSA expression using ImageJ software. Con, corn oil-injected (s.c.) + DW administration; BPH, TP (5 mg/kg, s.c.) + DW administration; BPH+K_EF, BPH+ heat-killed *E. faecalis* (7.5 × 10^12^ CFU/g, 2.21 mg/kg) administration; BPH+L_EF, BPH + live *E. faecalis* (1 × 10^11^ CFU/g, 166 mg/kg) administration; BPH+Fi, BPH + finasteride (1 mg/kg) administration. Data are expressed as mean ± S.E.M. (*n* = 8). ^##^*p* < 0.01 compared to Con. **p < 0.01 compared to BPH.

AR, 5AR2, and PSA are key proteins in the androgen signaling pathway. The expression levels of the 5AR2, ER, AR, and PSA proteins were significantly elevated in the prostate tissue from rats in the BPH group compared to that from rats in the Con group (Fig. 4). However, the prostate tissue from rats in the BPH+K_EF and BPH+L_EF groups showed a significant decrease in the expression of 5AR2, ER, AR, and PSA compared to those from rats in the BPH group.

### Effect of *E. faecalis* on the Expression of Growth Factors

**Fig. 5:**
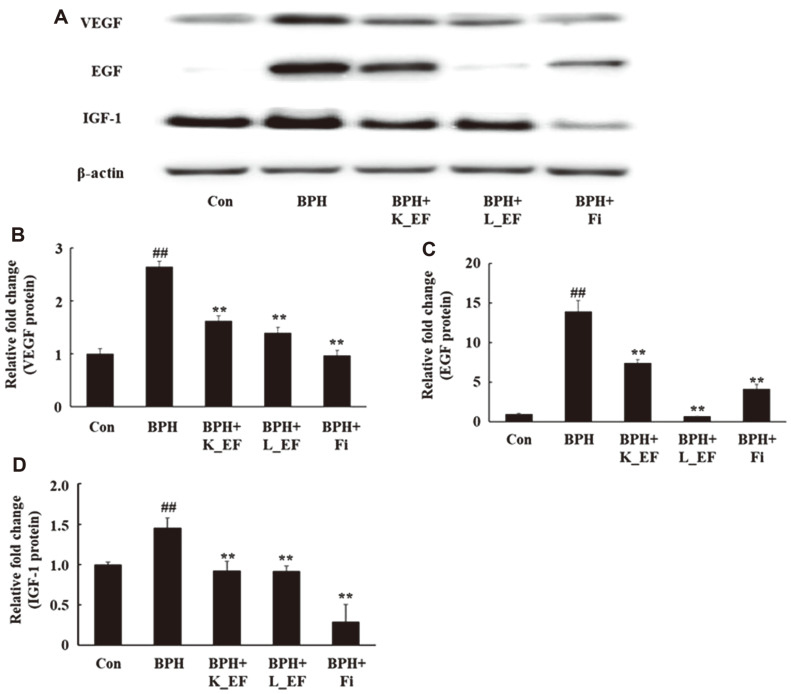
Effect of K_EF and L_EF on the expression of growth factors in prostate tissue from BPH rats. (**A**) Protein expression of VEGF, EGF and IGF-1. Densitometer analysis of (**B**) VEGF expression, (**C**) EGF expression (**D**) IGF-1 using ImageJ software. Con, corn oil-injected (s.c.) + DW administration; BPH, TP (5 mg/kg, s.c.) + DW administration; BPH+K_EF, BPH+ heat-killed *E. faecalis* (7.5 × 10^12^ CFU/g, 2.21 mg/kg) administration; BPH+L_EF, BPH + live *E. faecalis* (1 × 10^11^ CFU/g, 166 mg/kg) administration; BPH+Fi, BPH + finasteride (1 mg/kg) administration. Data are expressed as mean ± S.E.M. (*n* = 8). ^##^*p* < 0.01 compared to Con. *p < 0.05, **p < 0.01 compared to BPH.

In BPH, growth factors are over-expressed via androgen signaling, resulting in enhanced proliferation of prostate cells [25]. Consequently, the protein expression of VEGF, EGF, and IGF-1 in the prostate of BPH group were significantly increased compared to the Con group (Fig. 5, *p* < 0.01). On the contrary, BPH+K_EF and BPH+L_EF showed a significant reduction in the protein expression of the growth factors compared to the BPH group. BPH+Fi group also showed significantly reduced expression of VEGF, EGF and IGF-1 compared to the BPH group.

### Effect of *E. faecalis* on ERK Phosphorylation

**Fig. 6:**
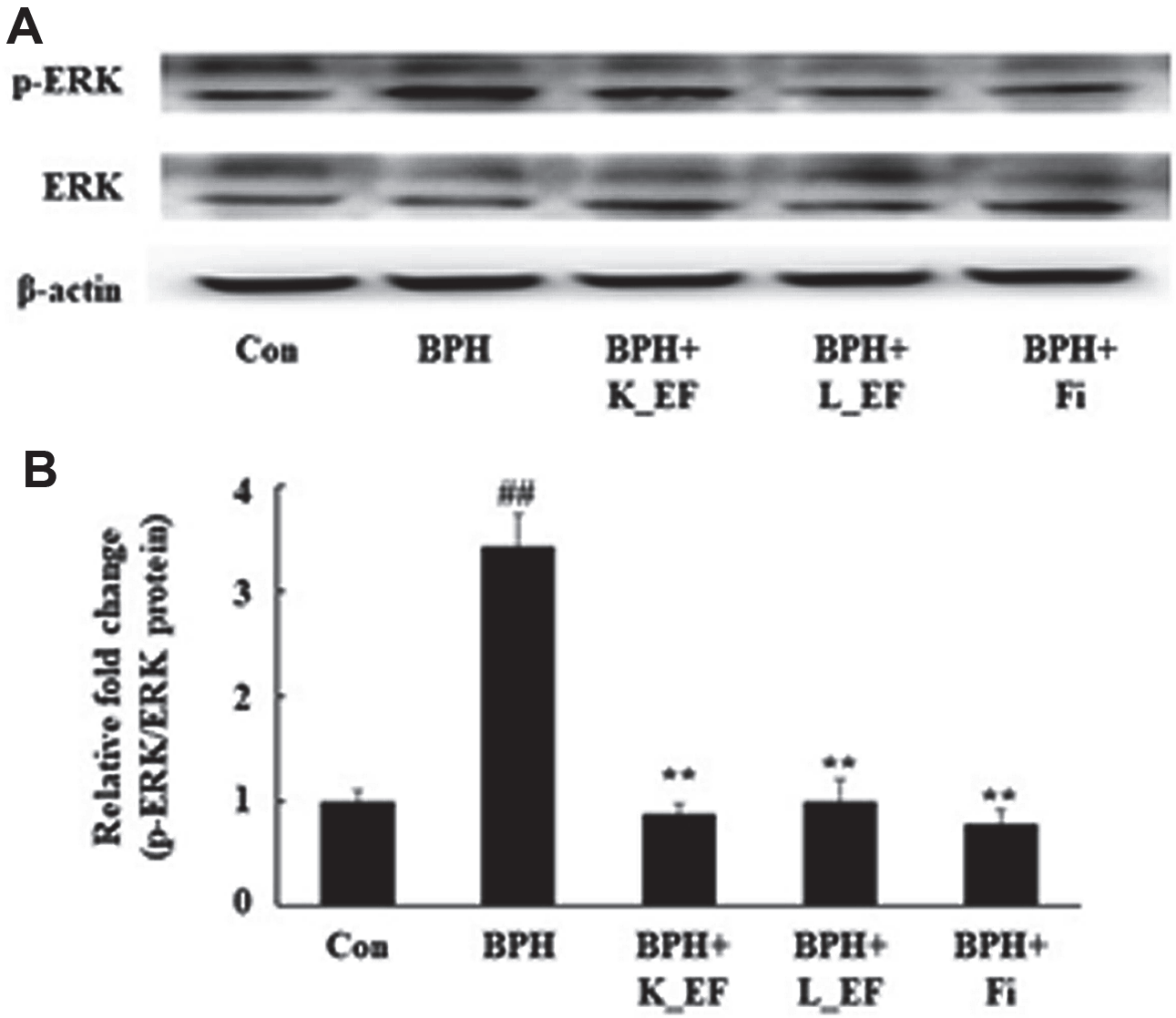
Effect of K_EF and L_EF on the phosphorylation of ERK in prostate tissue from BPH rats. (**A**) Protein expression of p-ERK and ERK. (**B**) Densitometer analysis of p-ERK/ERK ratio using ImageJ software. Con, corn oil-injected (s.c.) + DW administration; BPH, TP (5 mg/kg, s.c.) + DW administration; BPH+K_EF, BPH+ heat-killed *E. faecalis* (7.5 × 10^12^ CFU/g, 2.21 mg/kg) administration; BPH+L_EF, BPH + live *E. faecalis* (1 × 10^11^ CFU/g, 166 mg/kg) administration; BPH+Fi, BPH + finasteride (1 mg/kg) administration. Data are expressed as mean ± S.E.M. (*n* = 8). Significant differences at ^##^*p* < 0.01 compared to Con. *p < 0.05, **p < 0.01 compared to BPH.

The MAPK pathway is closely related to cell proliferation and apoptosis. In addition, increased growth factor expression by androgen signaling in prostate cells affects the ERK phosphorylation cascade [26]. Accordingly, Fig. 6 shows that ERK phosphorylation was significantly increased in the BPH group compared to the Con group. On the other hand, the phosphorylation of ERK was significantly reduced in the BPH+K_EF, BPH+L_EF, and BPH+Fi groups compared to the BPH group (Fig. 6).

### Effect of *E. faecalis* on the Expression of Proliferation and Genes

**Fig. 7:**
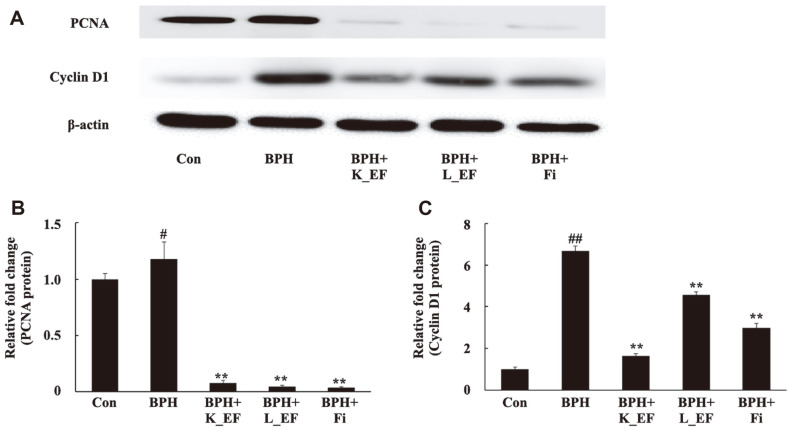
Effect of K_EF and L_EF on the expression of proliferation-related proteins in the prostate tissue from BPH rats. (**A**) Protein expression of PCNA and cyclin D1. Densitometer analysis of (**B**) PCNA expression and (**C**) Cyclin D1 expression using ImageJ software. Con, corn oil-injected (s.c.) + DW administration; BPH, TP (5 mg/kg, s.c.) + DW administration; BPH+K_EF, BPH+ heat-killed *E. faecalis* (7.5 × 10^12^ CFU/g, 2.21 mg/kg) administration; BPH+L_EF, BPH + live *E. faecalis* (1 × 10^11^ CFU/g, 166 mg/kg) administration; BPH+Fi, BPH + finasteride (1 mg/kg) administration. Data are expressed as mean ± S.E.M. (*n* = 8). ^#^*p* < 0.05, ^##^*p* < 0.01 compared to Con. *p < 0.05, **p < 0.01 compared to BPH.

The anti-proliferative efficacy of *E. faecalis* in BPH was analyzed. The expression of PCNA, a representative cell proliferation marker, and cyclin D1, a cell proliferation positive regulator, were measured [27, 28]. It was confirmed that PCNA and cyclin D1 were significantly over-expressed in the BPH group (Fig. 7). The results showed that oral administration of heat-killed *E. faecalis* or live *E. faecalis* significantly reduced the expression of PCNA and cyclin D1 (Fig. 7). Similarly, the expression of PCNA and cyclin D1 was significantly reduced in the BPH+Fi group.

### Effect of *E. faecalis* on the Expression of Apoptosis-Related Genes

**Fig. 8:**
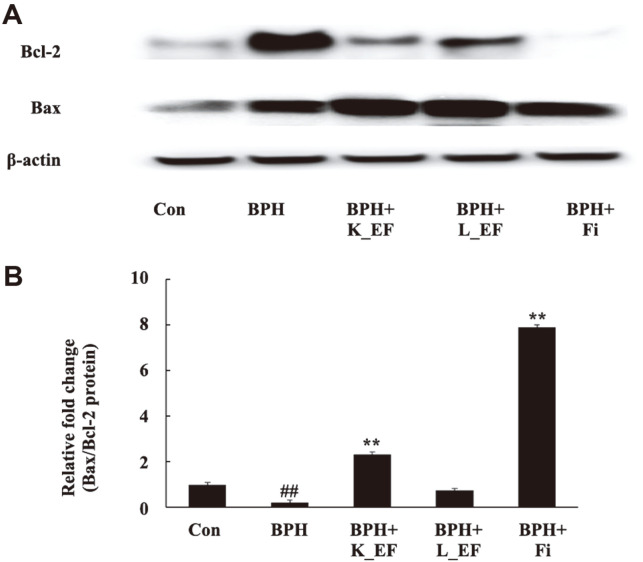
Effect of K_EF and L_EF on the expression of apoptosis-related proteins in prostate tissue from BPH rats. (**A**) Protein expression of Bcl-2 and Bax. (**B**) Densitometer analysis of Bax/Bcl-2 ratio using ImageJ software. Con, corn oil-injected (s.c.) + DW administration; BPH, TP (5 mg/kg, s.c.) + DW administration; BPH+K_EF, BPH+ heat-killed *E. faecalis* (7.5 × 10^12^ CFU/g, 2.21 mg/kg) administration; BPH+L_EF, BPH + live *E. faecalis* (1 × 10^11^ CFU/g, 166 mg/kg) administration; BPH+Fi, BPH + finasteride (1 mg/kg) administration. Data are expressed as mean ± S.E.M. (*n* = 8). ^#^*p* < 0.05 compared to Con. *p < 0.05, **p < 0.01 compared to BPH.

We sought to confirm whether the BPH alleviating effect of *E. faecalis* was due to apoptosis. It is known that the Bcl-2 family (Bcl-2, Bax) is a factor that affects the progression of apoptosis. Bcl-2 is an inhibitor of apoptosis, and Bax is a promoter of apoptosis [29]. In the BPH+K_EF group, the expression of Bcl-2 decreased while the expression of Bax increased compared to the BPH group (Fig. 8). BPH+Fi also showed that Bcl-2 levels were downregulated and that of Bax was upregulated. In the BPH+L_EF group, the expression of Bcl-2 was decreased and the BAX level was increased compared to the BPH group, but there was no significant difference.

## Discussion

BPH is a common chronic disease that causes bladder outlet obstruction (BOO) and lower urinary tract symptoms (LUTS) in men over 50 years old [30]. Currently, there is no complete treatment for BPH, and the commonly used treatment approaches involve 5-α reductase inhibitors and α-blockers. Since alpha blockers do not affect the prostate size, their use does not tackle the underlying problem of BPH [31]. Fi, a representative 5AR blocker, is the most effective drug used currently; however, it has been reported to produce several side effects. Therefore, there is increasing interest in developing a more effective and safe treatment strategy for BPH using natural products.

The prostate is a representative androgen-dependent organ. Biosynthesis of DHT from testosterone by 5AR2 is the most important factor in the prostate cell cycle [32]. DHT binds to AR and interacts with androgen response elements (AREs) at the promoter region of the growth factors and PSA, thereby enhancing the transcriptional activity of growth factors and PSA [33]. On the other hand, it has been known that PSA is not detected in animals other than humans. However, according to recent reports, studies have shown that anti-human PSA antibodies can recognize PSA-like proteins in the rat prostate [34]. There is experimental evidence that stimulation of ERα in the prostate affects the proliferative mechanism [35]. In addition, there was a report that the expression of ERα was increased in the BPH, and this was confirmed through this experiment [34]. Accordingly, we found that androgen signaling was over-expressed in BPH, and the expression levels of PSA and growth factor were also high. Growth factors are powerful mediators of matrix epithelial interactions and cell proliferation in the prostate. Therefore, growth factors can be used as indicators of BPH development [36]. Apoptosis is regulated by interactions between the Bcl-2 family. It is known that the pro-apoptotic effector, Bax, is inhibited by Bcl-2 and that the expression of Bcl-2 is increased in BPH.

We found that the administration of heat-killed *E. faecalis* and live *E. faecalis* downregulated androgen receptor signaling-related factors. Consequently, DHT level in the prostate tissue and serum was significantly reduced by heat-killed *E. faecalis* and live *E. faecalis*. Further, the decrease in binding of DHT and AR due to heat-killed *E. faecalis* or live *E. faecalis* administration significantly suppressed the expression of PSA and growth factors. It is speculated that the reduction of growth factor levels had a significant effect on EGFG/ERK signaling as well as reduction of cell proliferation and increase of apoptosis. ERK is known to be involved in cell growth and differentiation by being activated by growth factors, cytokines, and phorbol esters [26]. Therefore, we showed that heat-killed *E. faecalis* and live *E. faecalis* in BPH inhibited phosphorylation of ERK, resulting in decreased expression of PCNA and cyclin D1. In addition, heat-killed *E. faecalis* showed efficacy in increasing apoptosis in the prostate of BPH-induced rats by upregulating the expression of Bcl-2. Thus, live *E. faecalis* reduced the prostate index to 13.7% of the BPH group. Interestingly, compared to the BPH group, heat-killed *E. faecalis* reduced prostate weight by 17.1%, comparable to Fi (18.2%). In histological analysis using H&E staining, both heat-killed and live *E. faecalis* showed excellent efficacy in reducing Fi-like prostatic epithelial cell thickness.

In this study, administration of heat-killed and live *E. faecalis* was shown to help relieve an enlarged prostate. In addition, this study shows that not only live microorganism but also dead microorganism can be fully utilized as materials to relieve prostatic hyperplasia. Chronic inflammation has been reported in 75% of BPH patients [37]. Repetitive damage to the prostate tissue due to chronic inflammation causes compensatory proliferation of cells, which affects the expression of apoptosis-related proteins [38]. This leads to excessive tissue proliferation and increases the risk of BPH. Thus, chronic inflammation in the prostate tissue induces excessive cytokine secretion by T-cells, resulting in over-expression of growth factors in the prostate [39]. There have also been reports of high cytokine levels in BPH patients [40]. Previously, heat-killed *E. faecalis* has been shown to produce excellent effects in various inflammatory diseases [17, 18, 20]. Therefore, it is thought that *E. faecalis* can help relieve prostatic hyperplasia by alleviating inflammation in the prostate. In addition, heat-killed *E. faecalis* possesses the advantage that it can be used as food additives in a variety of foods because it is much more stable against heat processing and easy to store for a long time. Heat-killed *E. faecalis* can be added to various foods regardless of heat processing. For live *E. faecalis*, it can only be added to food without heat processing and must be under cold storage. Meanwhile, heat-killed *E. faecalis* is not affected by heat processing and cold storage. The heat-killed microorganisms are recognized as a raw material for natural products, and the range of application is wide, including general foods, health functional foods, and pharmaceuticals, so it is easy to use in a variety of ways [41]. However, the results to date do not show whether *E. faecalis* was directly responsible for androgen receptor signaling or whether it helped relieve enlarged prostate by reducing inflammatory factors. To understand these underlying molecular mechanisms, it is likely that further study of the metabolites of heat-killed *E. faecalis* and live *E. faecalis* is necessary.

## Conclusions

In this study, the administration of probiotics was shown to help alleviate prostatic hyperplasia. Furthermore, this study shows that not only live probiotics but also heat-killed probiotics can be fully utilized as functional food materials. There are currently only a few studies on the effects of probiotics on BPH. Therefore, the present study contributes to our understanding of the relationship between probiotic administration and prostatic hyperplasia.

## References

[1] Roehrborn CG (2005). Benign prostatic hyperplasia: an overview. Rev. Urol..

[2] Coffey DS, Walsh PC (1990). Clinical and experimental studies of benign prostatic hyperplasia. Urol. Clin. North Am..

[3] Farnsworth WE (1999). Estrogen in the etiopathogenesis of BPH. Prostate.

[4] Aryal M, Pandeya A, Bas BK, Lamsal M, Majhi S, Pandit R (2007). Oxidative stress in patients with benign prostate hyperplasia. JNMA J. Nepal Med. Assoc..

[5] Ozden C, Ozdal OL, Urgancioglu G, Koyuncu H, Gokkay S, Memis A (2007). The correlation between metabolic syndrome and prostatic growth in patients with benign prostatic hyperplasia. Eur. Urol..

[6] Awodele O, Adeyomoye AA, Awodele DF, Fayankinnu VB, Dolapo DC (2011). Cancer distribution pattern in south-western Nigeria. Tanzan. J. Health Res..

[7] McVARY KT, Rademaker A, Lloyd GL, Gann P (2005). Autonomic nervous system overactivity in men with lower urinary tract symptoms secondary to benign prostatic hyperplasia. J. Urol..

[8] Isaacs JT, Coffey DS (1989). Etiology and disease process of benign prostatic hyperplasia. Prostate.

[9] Geller J, Albert J, Lopez D, Geller S, Niwayama G (1976). Comparison of androgen metabolites in benign prostatic hypertrophy (BPH) and normal prostate. J. Clin. Endocrinol. Metab..

[10] Ho CK, Habib FK (2011). Estrogen and androgen signaling in the pathogenesis of BPH. Nat. Rev. Urol..

[11] Bostanci Y, Kazzazi A, Momtahen S, Laze J, Djavan B (2013). Correlation between benign prostatic hyperplasia and inflammation. Curr. Opin. Urol..

[12] Wermuth PJ, Del Galdo F, Jimenez SA (2009). Induction of the expression of profibrotic cytokines and growth factors in normal human peripheral blood monocytes by gadolinium contrast agents. Arthritis Rheum..

[13] Pierce KL, Tohgo A, Ahn S, Field ME, Luttrell LM, Lefkowitz RJ (2001). Epidermal growth factor (EGF) receptor-dependent ERK activation by G protein-coupled receptors: a co-culture system for identifying intermediates upstream and downstream of heparinbinding EGF shedding. J. Biol. Chem..

[14] Kerry RG, Patra JK, Gouda S, Park Y, Shin HS, Das G (2018). Benefaction of probiotics for human health: a review. J. Food Drug Anal..

[15] Wagner R, Pierson C, Warner T, Dohnalek M, Hilty M, Balish E (2000). Probiotic effects of feeding heat-killed *Lactobacillus acidophilus* and *Lactobacillus casei* to *Candida albicans*-colonized immunodeficient mice. J. Food Prot..

[16] Kang BS, Seo JG, Lee GS, Kim JH, Kim SY, Han YW (2009). Antimicrobial activity of enterocins from *Enterococcus faecalis* SL-5 against *Propionibacterium acnes*, the causative agent in acne vulgaris, and its therapeutic effect. J. Microbiol..

[17] Choi EJ, Iwasa M, Han KI, Kim WJ, Tang Y, Hwang YJ (2016). Heat-killed *Enterococcus faecalis* EF-2001 ameliorates atopic dermatitis in a murine model. Nutrients.

[18] Choi EJ, Iwasa M, Han KI, Kim WJ, Tang Y, Han WC (2016). Effect of *Enterococcus faecalis* EF-2001 on experimentally induced atopic eczema in mice. Food Sci. Biotechnol..

[19] Chang SJ, Lee MH, Kim WJ, Chae Y, Iwasa M, Han KI (2019). Effect of heat-killed *Enterococcus faecalis*, EF-2001 on C2C12 myoblast damage induced by oxidative stress and muscle volume decreased by sciatic denervation in C57BL/6 mice. J. Life Sci..

[20] Choi EJ, Lee HJ, Kim WJ, Han KI, Iwasa M, Kobayashi K (2019). *Enterococcus faecalis* EF-2001 protects DNBS-induced inflammatory bowel disease in mice model. PLoS One.

[21] Gu YH, Choi H, Yamashita T, Kang KM, Iwasa M, Lee MJ (2017). Pharmaceutical production of anti-tumor and immunepotentiating *Enterococcus faecalis*-2001 β-glucans: enhanced activity of macrophage and lymphocytes in tumor-implanted mice. Curr. Pharm. Biotechnol..

[22] Choi YJ, Fan M, Tang Y, Yang HP, Hwang JY, Kim EK (2019). In vivo effects of polymerized anthocyanin from grape skin on benign prostatic hyperplasia. Nutrients.

[23] Ohtake A, Ukai M, Saitoh C, Sonoda R, Noguchi Y, Okutsu H (2006). Effect of tamsulosin on spontaneous bladder contraction in conscious rats with bladder outlet obstruction: comparison with effect on intraurethral pressure. Eur. J. Pharmacol..

[24] Sheth SG, Flamm SL, Gordon FD, Chopra S (1998). AST/ALT ratio predicts cirrhosis in patients with chronic hepatitis C virus infection. Am. J. Gastroenterol..

[25] Soulitzis N, Karyotis I, Delakas D, Spandidos DA (2006). Expression analysis of peptide growth factors VEGF, FGF2, TGFB1, EGF and IGF1 in prostate cancer and benign prostatic hyperplasia. Int. J. Oncol..

[26] McCubrey JA, Steelman LS, Chappell WH, Abrams SL, Wong EW, Chang F (2007). Roles of the Raf/MEK/ERK pathway in cell growth, malignant transformation and drug resistance. Biochim. Biophys. Acta.

[27] Dietrich DR (1993). Toxicological and pathological applications of proliferating cell nuclear antigen (PCNA), a novel endogenous marker for cell proliferation. Crit. Rev. Toxicol..

[28] Tashiro E, Tsuchiya A, Imoto M (2007). Functions of cyclin D1 as an oncogene and regulation of cyclin D1 expression. Cancer Sci..

[29] Vela-Navarrete R, Escribano-Burgos M, Farre AL, Garcia-Cardoso J, Manzarbeitia F, Carrasco C (2005). Serenoa repens treatment modifies bax/bcl-2 index expression and caspase-3 activity in prostatic tissue from patients with benign prostatic hyperplasia. J. Urol..

[30] Dmochowski RR (2005). Bladder outlet obstruction: etiology and evaluation. Rev. Urol..

[31] Boyle P, Roehrborn C, Harkaway R, Logie J, de La Rosette J, Emberton M (2004). 5-Alpha reductase inhibition provides superior benefits to alpha blockade by preventing AUR and BPH-related surgery. Eur. Urol..

[32] Nickel JC (2008). Inflammation and benign prostatic hyperplasia. Urol. Clin. North Am..

[33] Sciarra A, Mariotti G, Salciccia S, Gomez AA, Monti S, Toscano V (2008). Prostate growth and inflammation. J. Steroid Biochem. Mol. Boil..

[34] Chughtai B, Lee R, Te A, Kaplan S (2011). Role of inflammation in benign prostatic hyperplasia. Rev. Urol..

[35] Penna G, Mondaini N, Amuchastegui S, Degli Innocenti S, Carini M, Giubilei G (2007). Seminal plasma cytokines and chemokines in prostate inflammation: interleukin 8 as a predictive biomarker in chronic prostatitis/chronic pelvic pain syndrome and benign prostatic hyperplasia. Eur. Urol..

[36] Bechis SK, Otsetov AG, Ge R, Olumi AF (2014). Personalized medicine for the management of benign prostatic hyperplasia. J. Urol..

[37] Youn DH, Park J, Kim HL, Jung Y, Kang J, Jeong MY (2017). Chrysophanic acid reduces testosterone-induced benign prostatic hyperplasia in rats by suppressing 5α-reductase and extracellular signal-regulated kinase. Oncotarget.

[38] Ellem SJ, Risbridger GP (2009). The dual, opposing roles of estrogen in the prostate. Ann. NY Acad. Sci..

[39] Lee KL, Peehl DM (2004). Molecular and cellular pathogenesis of benign prostatic hyperplasia. J. Urol..

[40] Dejean LM, Martinez-Caballero S, Manon S, Kinnally KW (2006). Regulation of the mitochondrial apoptosis-induced channel, MAC, by BCL-2 family proteins. Biochim. Biophys. Acta.

[41] Choi MS, Chang SJ, Chae Y, Lee MH, Kim WJ, Iwasa M (2018). Anti-inflammatory effect of heat-killed *Enterococcus faecalis*, EF-2001. J. Life Sci..

